# A Three-Dimensional Infinite Collapse Map with Image Encryption

**DOI:** 10.3390/e23091221

**Published:** 2021-09-17

**Authors:** Wenhao Yan, Zijing Jiang, Xin Huang, Qun Ding

**Affiliations:** Electronic Engineering College, Heilongjiang University, Harbin 150080, China; yanwh0512@163.com (W.Y.); 1213046@hlju.edu.cn (Z.J.); 1202875@hlju.edu.cn (X.H.)

**Keywords:** chaos, dynamic characteristics, image encryption, 3D infinite collapse map

## Abstract

Chaos is considered as a natural candidate for encryption systems owing to its sensitivity to initial values and unpredictability of its orbit. However, some encryption schemes based on low-dimensional chaotic systems exhibit various security defects due to their relatively simple dynamic characteristics. In order to enhance the dynamic behaviors of chaotic maps, a novel 3D infinite collapse map (3D-ICM) is proposed, and the performance of the chaotic system is analyzed from three aspects: a phase diagram, the Lyapunov exponent, and Sample Entropy. The results show that the chaotic system has complex chaotic behavior and high complexity. Furthermore, an image encryption scheme based on 3D-ICM is presented, whose security analysis indicates that the proposed image encryption scheme can resist violent attacks, correlation analysis, and differential attacks, so it has a higher security level.

## 1. Introduction

The 21st century is a new era of leapfrog development of information technology. Information technology, led by big data, artificial intelligence (AI), and computer network technology, has become a key factor in the development of a country’s political, military, economic, cultural, and educational undertaking. We usually use digital images as a widely used data format, since it carries a great amount of information in a visualized manner [1]. Billions of digital images are stored, copied, and transmitted every day through third-party platforms or insecure channels. An increasing attention is being paid by researchers to image security [2,3,4]. Some work such as information hiding, watermarking, and image encryption is done to protect the security of digital images [5,6]. Among them, image encryption is the most direct way to transform the plaintext image into noise-like information. 

Chaos is the inherent randomness of deterministic system and a special motion of nonlinear dynamic system, which exists widely in nature. The application of chaos in cryptography has become a hot research field, owing to its unpredictability and sensitivity to initial values. Chaotic systems can generate pseudo-random sequences with low correlation and high complexity [7,8,9,10,11,12,13,14,15]. An image encryption scheme generally contains two parts: confusion and diffusion. The confusion characteristic is obtained by randomly separating the adjacent pixels of the plaintext image, and the diffusion characteristic is obtained by diffusing the slight differences of the plaintext image to all the pixels of the ciphertext image. Fan et al. [16] proposed a new image encryption scheme, and a self-synchronous chaotic stream cipher was applied to the new scheme. Alawida et al. presented an image encryption based on a hybrid digital chaotic system in 2019 [17]. Alvarez et al. proposed some basic requirements for cryptosystems based on chaos [18]. The key sequences generated by chaotic systems exhibit many excellent cryptographic properties. In addition, the method of applying chaos to cryptography is easy to realize, and the algorithm has fast processing speed, large key space, and high security, which is very suitable for processing large amounts of data and greatly simplifies the design process of traditional sequential cryptography [19,20]. In an encryption scheme based on chaos, the security level mainly relies on the complexity of chaotic maps. However, the initial conditions and orbits of 1D chaotic maps can be easily predicted due to their simple trajectories and few variables [21,22]. Furthermore, when the system parameters are in a certain interval, the chaotic dynamics behavior will weaken or disappear [23,24]. In order to enhance the dynamic characteristics and complexity of the chaotic system, several HD chaotic maps with hyperchaotic properties were proposed [25,26]. However, some HD chaotic maps exhibit weak chaotic performance in certain intervals [14,23,24,26]. Thus, it makes sense to design a chaotic map with unpredictable and robust chaotic performance.

The contributions and novelties of this paper are summarized as follows: (1) a three-dimensional (3D) hyperchaotic map, called the 3D infinite collapse map (3D-ICM), is proposed in this paper. Quantitative evaluation criteria are used to study the chaotic characteristics of 3D-ICM, such as the Lyapunov exponent and Sample entropy. The results of the evaluation criteria show that 3D-ICM shares more complex chaotic dynamical behaviors than existing 3D chaotic maps. (2) We also propose an image encryption scheme based on the 3D-ICM. Both confusion and diffusion operations are based on sequences produced by the 3D-ICM. The simulation results show that the scheme can efficiently encrypt various types of images. Furthermore, different encryption schemes to encrypt the same image were given. Compared with other schemes, this scheme has faster encryption speed. Finally, security analysis shows that the scheme can also resist various attacks including brute-force attack, statistic attack, correlation analysis, and differential attack, which indicates the image encryption scheme has a high level of security. 

The rest of this paper is organized as follows. The 3D-ICM and the evaluation of its chaotic performance are presented in Section 2. Section 3 introduces an image encryption scheme and gives the simulation results of different images. Section 4 analyzes the security of the proposed image encryption scheme. Finally, some conclusions are drawn in Section 5.

## 2. The 3D Infinite Collapse Map

In order to overcome the above weakness of the low dimensional discrete-time chaotic map, linear combinations of the output values of existing chaotic systems are proposed to enhance the chaotic characteristics in [13,14]. Because the linear combination cannot change the output value of the original system, only the linear combination of these values, the performance of the presented system is not very good. Thus, a great deal of literature has been devoted to the study of the nonlinear transformations of chaotic output sequences [7,8,21,24,25,26]. However, these transformations are based on 1D and 2D chaotic systems. In order to further enhance its chaotic characteristics, this section mainly introduces the 3D infinite collapse map (3D-ICM), and the chaos characteristics are studied from the following three aspects: (1) the attractor; (2) the Lyapunov exponent (LE); (3) Sample Entropy (SE).

### 2.1. Mathematical Definition

An infinite collapse map (ICM) was introduced [27], and the mathematical definition of 1D-ICM is as follows:

(1) xi+1=sinaxi,

where the control parameter is a≠0, and x is the state variable of the system. One-dimensional chaotic systems can be easily predicted by implementing some estimation technologies due to its simple structure. A 2D-ICM integrates two 1D-ICMs with different parameters [28]. In order to further enhance the complexity of chaotic systems, a 3D-ICM is proposed. The mathematical definition of 3D-ICM is as follows:

(2) xi+1=sinaxisinbyisincziyi+1=sinczisinbyi    zi+1=sincyisinbzi    ,

where a, b, and c are control parameters of the system; x, y, and z are the state variables of the system. In this paper, a, b, c∈ℝ and a≠0, b≠0,c≠0. As shown in Formula (2), 3D-ICM consists of three 1D-ICMs with different system parameters. When a=0, the mathematical definition of 3D-ICM is the same as that of 2D-ICM. Thus, 2D-ICM is a special case of 3D-ICM.

### 2.2. Performance Evaluation

Several measures about chaotic maps, including the attractor, LE, and Sample Entropy (SE), are adopted to evaluate chaotic properties of 3D-ICM. Furthermore, the proposed 3D-ICM is compared with two existing 3D chaotic maps, i.e., 3D discrete hyperchaotic systems (3D-DHCS) [29], and a 3D Henon map [30]. In addition, it is compared with two existing chaotic maps, i.e., 1D-ICM and 2D-ICM.

#### 2.2.1. Attractor

**Figure 1:**
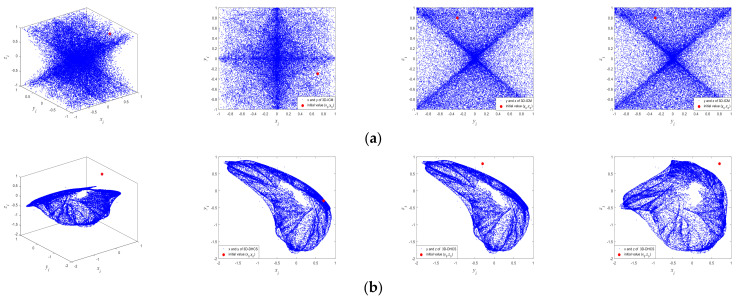

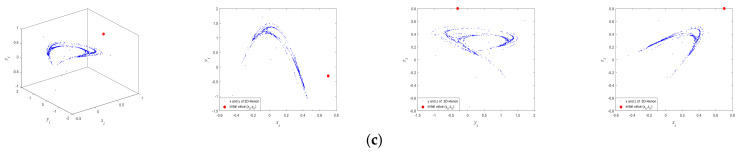
Attractors of 3D chaotic maps: (**a**) 3D-ICM; (**b**) 3D-DHCS; (**c**) 3D-Henon.

The phase diagram of a chaotic system refers to a set of numbers to which the system can evolve under given initial values. In the case of 3D chaotic systems, their attractors can be characterized by a larger number of points occupying a region in a three-dimensional phase space. To visualize the attractors of 3D chaotic systems, the initial value $\left(0.7,\;-0.3,\;0.8\right)$ and iteration times i=20,000 are selected. A comparison of chaotic attractors of 3D-ICM and other 3D chaotic maps is given in Figure 1. As is depicted in Figure 1, the output sequence of 3D-ICM almost fills the entire phase space range of $\left(-1,\;1\right),$ which shows that 3D-ICM has the better ergodicity property than 3D-DHCS and 3D-Henon.

#### 2.2.2. Lyapunov Exponent

In the theory of nonlinear dynamics, the Lyapunov exponent (LE) is an important chaotic characteristic that is used to describe the infinitesimal deviation of orbit in phase space by a quantitative method. The sensitivity to initial conditions is an important characteristic of chaos, that is, two orbits in phase space that are close to each other will separate exponentially over time. The LE represents a measure of the mean convergence or mean divergence of similar orbitals in phase space. The larger the value of LE is, the faster the phase space trajectory diverges. This means that the more sensitive it is to the initial conditions, the more chaotic the system is. The Lyapunov exponents of an n-dimensional chaotic system is calculated as follows: Let the Jacobian matrix of n-dimensional chaotic system be J. Given the initial value $\left(x_{1}(0),\;x_{2}\left(0\right),\;\cdots ,\;x_{k}\left(0\right)\right),$ and we can obtain a series of values ${\left\{\left(x_{1}(i),\;x_{2}\left(i\right),\;\cdots ,\;x_{k}\left(i\right)\right)\right\}}_{i=1}^{k}.$ The Jacobian matrix of the first *n* is as follows:

(3) $$\left\{\begin{array}{c} J_{0}=J\left(x_{1}\left(0\right),\;x_{2}\left(0\right),\;\cdots ,\;x_{k}\left(0\right)\right)\;\;\;\;\\ J_{1}=J\left(x_{1}\left(1\right),\;x_{2}\left(1\right),\;\cdots ,\;x_{k}\left(1\right)\right)\;\;\;\;\;\;\\ \cdots \\ J_{k-1}=J\left(x_{1}\left(n-1\right),\;x_{2}\left(n-1\right),\;\cdots ,\;x_{k}\left(k-1\right)\right) \end{array}\right..$$

$J_{k}$ can be obtained from the following equation:

(4) $$J_{k}=J_{0}J_{1}\cdots J_{k-1}$$

The Lyapunov exponents of an n-dimensional chaotic system can be obtained as follows:

(5) $$LE_{1}=\underset{k\to \infty }{\lim}\frac{1}{k}\ln\left|\lambda_{1}\right|,\;LE_{2}=\underset{k\to \infty }{\lim}\frac{1}{k}\ln\left|\lambda_{2}\right|,\;\cdots ,\;LE_{n}=\underset{k\to \infty }{\lim}\frac{1}{k}\ln\left|\lambda_{n}\right|,$$

where $\lambda_{1}\;\lambda_{2}\;,\cdots ,\;\lambda_{k}$ are the eigenvalues of matrix $J_{k}$.

**Figure 2:**
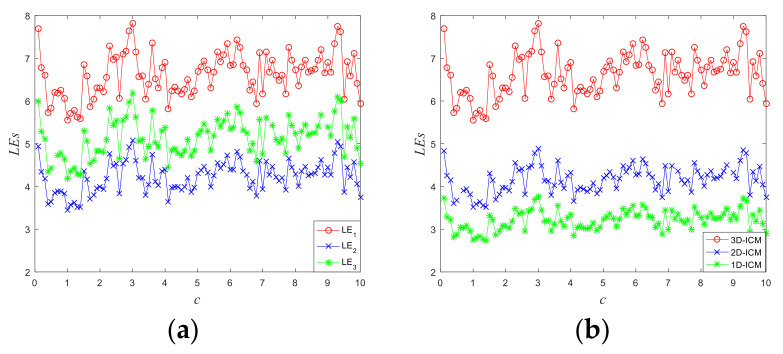
The values of the Lyapunov exponent of chaotic maps: (**a**) the three Les of 3D-ICM; (**b**) comparison of Les between 3D-ICM and other ICM.

As Figure 2a illustrates, the three Les of the 3D-ICM are greater than 0 in all parameter spaces. From the attractor in the last subsection, we know that the 3D-ICM is globally bounded. What is more, the three Les are all greater than 0 in this subsection, thus the 3D-ICM is a hyperchaotic system. As is depicted in Figure 2b, the LE of the 3D-ICM is larger than the Les of the other ICM, which indicates that the 3D-ICM is chaotic map with more complex chaotic dynamical behaviors.

#### 2.2.3. Sample Entropy

**Figure 3:**
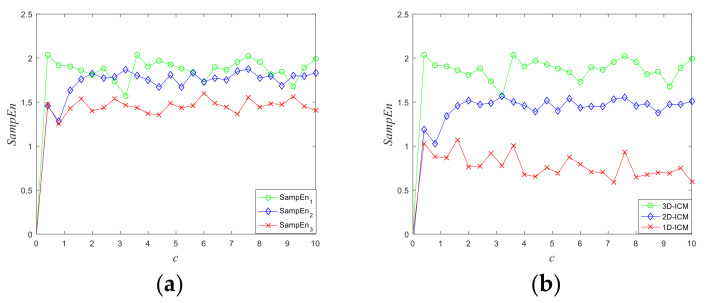
The values of Sample entropy of chaotic maps: (**a**) the three Ses of 3D-ICM; (**b**) comparison of Ses between 3D-ICM and other chaotic maps.

At present, the approximate entropy (ApEn) Algorithm [31] is widely used to measure the complexity of chaotic sequences. However, since the ApEn algorithm avoids errors by counting the number of templates that match its own data, if the threshold value is small, there will be a large number of template matches, resulting in the phenomenon that the effect of deviation is not obvious, so there is a margin for error. In 2000, a new quantization algorithm of time series complexity, called Sample Entropy (SE), was proposed [32], which is an improved algorithm of ApEn. The SE of a time series $\left\{x_{1},\;x_{2},\;\cdots ,\;x_{N}\right\}$ is defined by as follows:

(6) $$SE\left(m,r,N\right)=-\log\frac{A}{B},$$

where dimension m and distance r are usually set as 2 and 0.2×SD, respectively. *SD* represents the standard deviation of the tested time series, and A and B are the number of vectors, which are $d\left[X_{m+1}(i),\;X_{m+1}(j)\;\right]<r$ and $d\left[X_{m}(i),\;X_{m}(j)\;\right]<r,$ respectively. The template vectors $X_{m}\left(i\right)\;=\;\left\{x_{i},\;x_{i+1},\;\cdots ,\;x_{i+m-1}\right\},$ and $d\left[X_{m}(i),\;X_{m}(j)\;\right]$ are the Chebyshev distance between $X_{m}\left(i\right)$ and $X_{m}\left(j\right).$ As Figure 3a illustrates, the three Ses of the 3D-ICM are greater than 0 in all parameter spaces. Figure 3b compares the Ses of existing chaotic maps. It can be observed that the 3D-ICM has much larger Ses than others, which indicates 3D-ICM has more complex output sequences. 

It can be known from the above analysis that the trajectories of the 3D-ICM are difficult to predict over time owing to its complex chaotic properties. Which indicates that the 3D-ICM shares a much larger region, better ergodicity, and more unpredictable chaotic behaviors than others in terms of the results of the attractor, LE, and SE. In the next section, the 3D-ICM will be applied in image encryption.

## 3. An Image Encryption Scheme Based on 3D-ICM

**Figure 4:**
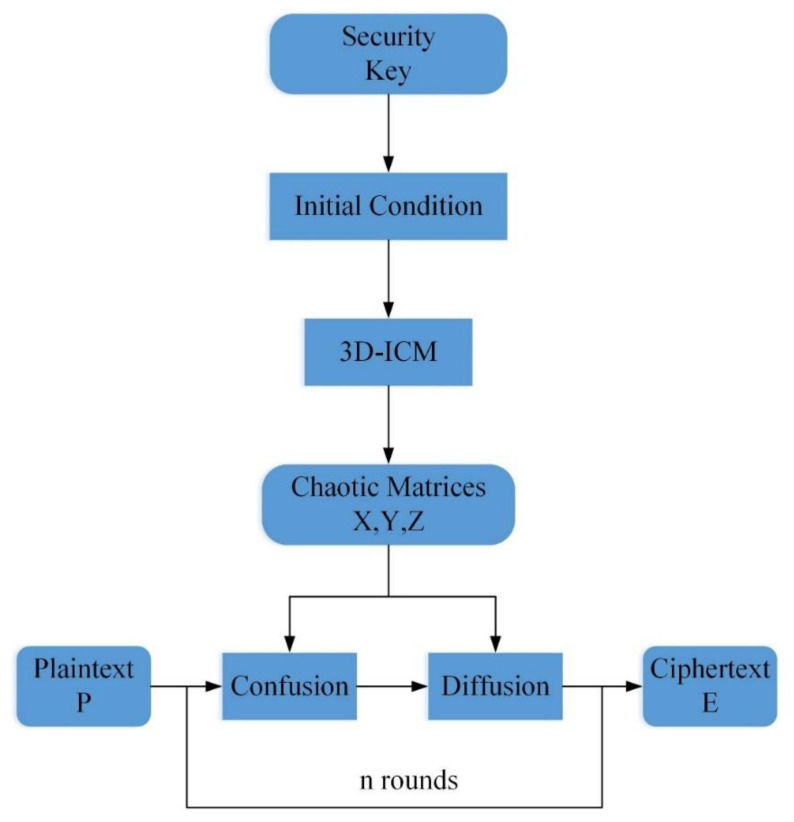
Structure of the proposed image encryption algorithm.

An image encryption scheme based on the 3D-ICM is presented in this section. The structure of the image encryption scheme is shown in Figure 4. The security key produces the initial conditions for the 3D-ICM to generate a chaotic output sequence. The proposed scheme is mainly based on the basic concepts of confusion and diffusion. The confusion part can effectively separate adjacent pixels of an image into different positions, while the diffusion part can change the pixels’ values using a reversible transform. Multiple rounds of confusion and diffusion were carried out to obtain a higher level of security. In this paper, two rounds of confusion and diffusion are used to compromise security and computational efficiency. The decryption process is the inverse of the encryption process. As for the color image, we first only need to divide the color image into three channels of R, G, and B, and then perform confusion and diffusion processing on these three channels. We then only need to recombine the three encrypted channels to obtain the result: the encrypted color image. Decryption is the reverse process of encryption.

### 3.1. Key Distribution

**Figure 5:**
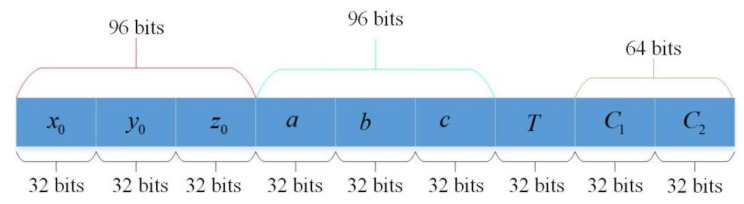
The structure of the security key.

The initial conditions of the 3D-ICM are determined by the security key. When the key space of the cryptosystem based on chaotic maps is more than $2^{100}$, it can resist brute-force attacks [29]. The algorithm’s key length is set to 256 bits in this paper, so the algorithm’s key space is $2^{256},$ which demonstrates it can resist brute-force attacks. Figure 5 illustrates the structural framework of the security key. We can see from Figure 5 that it contains nine parts $\left\{a,\;b,\;c,\;x_{0},\;y_{0},\;z_{0},\;T,\;C_{1},\;C_{2}\right\},$ where $\left\{a,\;b,\;c,\;x_{0},\;y_{0},\;z_{0}\right\}$ are the initial states, T is the perturbation parameter in order to disturb the initial conditions, and $C=\left\{C_{1},\;C_{2}\right\}$ contains two coefficients for the perturbation parameter. Each parameter $a,\;b,\;c,\;x_{0},\;y_{0},\;z_{0},\;T$, $\;C_{1},\;C_{2}$ has a length of 32 bits. The 32-bit binary strings in the security key $\left\{s_{1},\;s_{2},\;\cdots ,\;s_{40}\right\}$ are used to produce decimal 9 parameters using the IEEE 754 format. Thus, the initial conditions of the 3D-ICM for the two rounds can be calculated as follows:

(7) $$\left\{\begin{array}{c} x_{0}^{\left(i\right)}=\left(x_{0}+T\times C_{i}\right)\mathrm{mod}1\\ y_{0}^{\left(i\right)}=\left(y_{0}+T\times C_{i}\right)\mathrm{mod}1\\ z_{0}^{\left(i\right)}=\left(z_{0}+T\times C_{i}\right)\mathrm{mod}1\\ a_{0}^{\left(i\right)}=\left(a_{0}+T\times C_{i}\right)\;\;\;\\ b_{0}^{\left(i\right)}=\left(b_{0}+T\times C_{i}\right)\;\;\;\\ c_{0}^{\left(i\right)}=\left(c_{0}+T\times C_{i}\right)\;\;\; \end{array}\right..$$

### 3.2. Confusion Part

A novel confusion method, using three chaotic matrices to randomly separate the adjacent pixels of an image into different positions, is presented in this part. The confusion operation should be carried out in a square matrix, $L^{2}\times L^{2}$, where parameter L is the block size. If the plaintext image which will be processed has a size X×Y, the length of $L^{2}$ is obtained as follows:

(8) $$L^{2}=\min\left\{X,\;Y\right\}.$$

The detail confusion process algorithm is described in Algorithm 1.
**Algorithm 1.** The confusion process of the proposed image encryption scheme.**Input:** The plaintext image P and three initial values $\left\{x\left(0\right),\;y\left(0\right),\;z\left(0\right)\right\}.$**Output:** The confusion image F.Truncate the plaintext image as size $L^{2}\times L^{2},$ where $L^{2}$ is calculated using Equation (8).2.Generate three chaotic sequences, *X*, *Y*, and *z*, where these lengths are $L^{2}\times L^{2}.$3.Reshape the sequences *X*, *Y*, and *z* in columns into $L^{2}\times L^{2}.$ matrices, denoted as *X_L_*, *Y_L_*, and *Z_L_*.4.Matrices $S1=X_{L}\times Y_{L}$$\;\mathrm{and}\;S2=X_{L}\times Z_{L}$ can be obtained.5.Sort $S_{1}$ and $S_{2}$ in ascending order, and obtain their index vectors $I_{1}$ and $I_{2}$.6.The pixel locations of the plaintext image *P* are rearranged using the index matrix *I_i_*, where *i* = {1,2}.7.The confusion image *F* is obtained.

**Figure 6:**
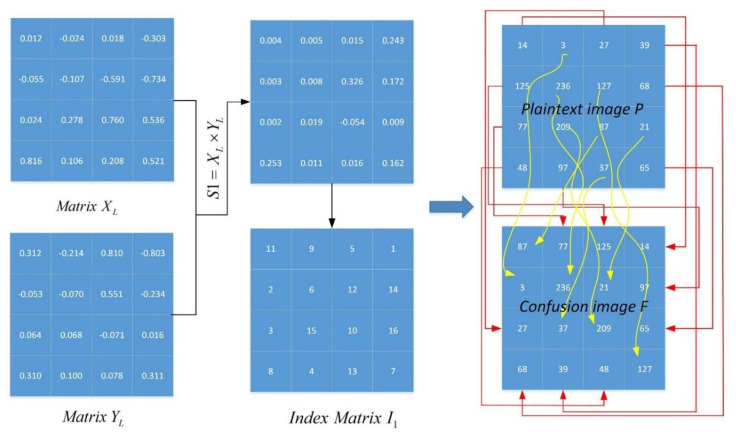
A numerical example of the confusion process.

**Figure 7:**
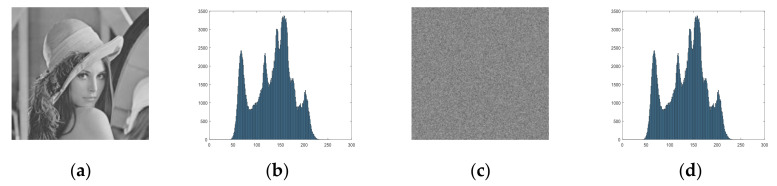
Confusion results: (**a**) Lena image; (**b**) histogram of (**a**); (**c**) confusion image; (**d**) histogram of (**c**).

A numerical example is presented in Figure 6. Matrices $X_{L}$ and $X_{L}$ are reshaped by the chaotic sequences X and Y, whose length are $4^{2}.$ It can be observed that almost every pixel is scrambled after a round confusion. Figure 7 presents a comparison plaintext image P and confusion image F. The histogram of F is the same as that of P due to the confusion process only changing the positions of the image’s pixels.

### 3.3. Diffusion Part

**Figure 8:**
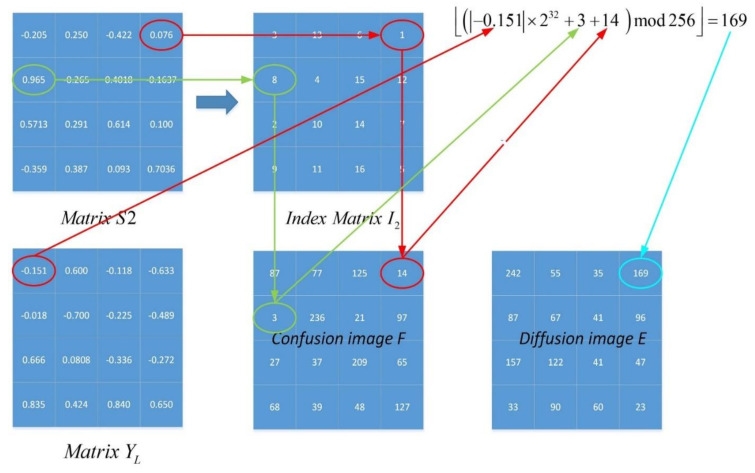
A numerical example of the diffusion process.

**Figure 9:**
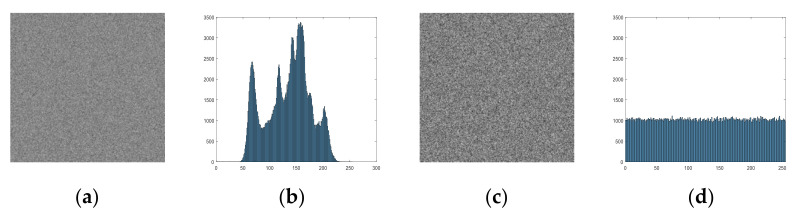
Diffusion results: (**a**) confusion image; (**b**) histogram of (**a**); (**c**) diffusion image; (**d**) histogram of (**c**).

By changing the value of pixel points, the diffusion part achieves the result that the small differences in the plaintext image are diffused to almost all pixels of the ciphertext image. The chosen plaintext attack is used to attack an encryption scheme via examining how a small difference affects the encryption performances of a cryptosystem. An excellent diffusion part can help cryptosystems to defeat the attack. To obtain a much higher level of security, a diffusion scheme relying on an index matrix related to chaotic sequence is presented. In one round of encryption, the matrix $S_{1}$ and its index matrix $I_{1}$ are used for confusion, firstly, while the other matrix $S_{2}$ and its index matrix $I_{2}$ are used for diffusion. Figure 8 shows a numerical example of the scheme. Suppose that confusion result F, chaotic matrix $Y_{L}$, and the current pixel can randomly be changed using the previous one and the chaotic sequence. The pixel value of diffusion image can be obtained by:

(9) $$D_{i}=\left\{\begin{array}{c} \left\lfloor \left(F_{i}+F_{M\times N}+\left|Y(i)\right|\times 2^{32}\right)\mathrm{mod}256\right\rfloor ,\;\;\;\;\;\;\;\;i=1\\ \left\lfloor \left(F_{i}+D_{i-1}+\left|Y(i)\right|\times 2^{32}\right)\mathrm{mod}256\right\rfloor ,\;\;\;i\in \left[2,M\times N\right] \end{array}\right.$$

where $\left\lfloor \alpha \right\rfloor$ is the floor operation to obtain the greatest integer, which is not larger than α. As Figure 9 illustrates, the histogram of the diffuse image is evenly distributed, which is completely different from the plaintext image and the confused image. In the first round of encryption, the matrix $S_{1}$ and its index matrix $I_{1}$ are used for confusion, while the other matrix $S_{2}$, its index matrix $I_{2}$, and chaotic sequence Y are used for diffusion. Furthermore, in the second round of encryption, the matrix $S_{2}$ and its index matrix $I_{2}$ are used for confusion, while the other matrix $S_{1}$, its index matrix $I_{1},$ and chaotic sequence Z are used for diffusion. The two rounds of confusion and diffusion operations are applied to the proposed image encryption scheme to obtain the final image. The decryption process is generally the inverse operation of the encryption process. Thus, the process of diffusion can be described as follows:

(10) $$F_{i}=\left\{\begin{array}{c} \left\lfloor \left(D_{i}-D_{i-1}-\left|Y(i)\right|\times 2^{32}\right)\mathrm{mod}256\right\rfloor \;\;\;i\in \left[2,M\times N\right],\\ \left\lfloor \left(D_{i}-F_{M\times N}-\left|Y(i)\right|\times 2^{32}\right)\mathrm{mod}256\right\rfloor \;\;\;\;\;\;\;i=1. \end{array}\right.$$

What is more, the original image can be obtained using the inverse operation of confusion.

### 3.4. Simulation Results

**Figure 10:**
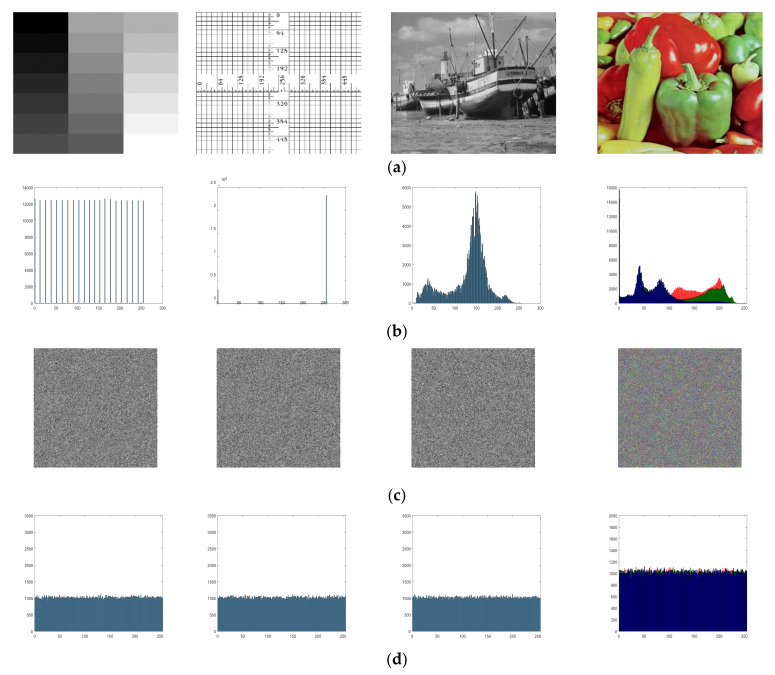
Simulation results: (**a**) plaintext images; (**b**) histograms of plaintext images; (**c**) encryption results of plaintext images; (**d**) histogram of the encryption results of plaintext images.

In an image encryption scheme, different types of images should be encrypted into ciphertext images with a high security level. Figure 10 presents the different types of images encrypted by the proposed method. All plaintext images include three grayscale images and a color image. These ciphertext images are random-like images with uniformly distributed, which indicates the proposed method can effectively encrypt different types of images. In addition, an image encryption scheme should have high encryption efficiency. The proposed encryption scheme can achieve a higher encryption efficiency owing to confusion and diffusion having lower computational complexity. The complete numerical experiments are performed in Matlab R2018a in a workstation with Intel(R) Core (TM) i7-1180H CPU @ 2.3 GHz with 16.0 GB RAM memory under Windows 10 OS. Table 1 compares the required time between the proposed encryption scheme and existing encryption schemes in encrypting same image. Here, images in USC-SIPI Miscellaneous dataset are used in this paper. It can be observed that the proposed encryption scheme has faster encryption speeds than existing encryption schemes for the same image. Therefore, the proposed method exhibits lower time complexity.

## 4. Security Analysis

Some analysis such as key security analysis, histogram analysis, correlation analysis, and differential attack, are presented to indicate the even better performance of the proposed image encryption scheme.

### 4.1. Key Security Analysis

**Figure 11:**
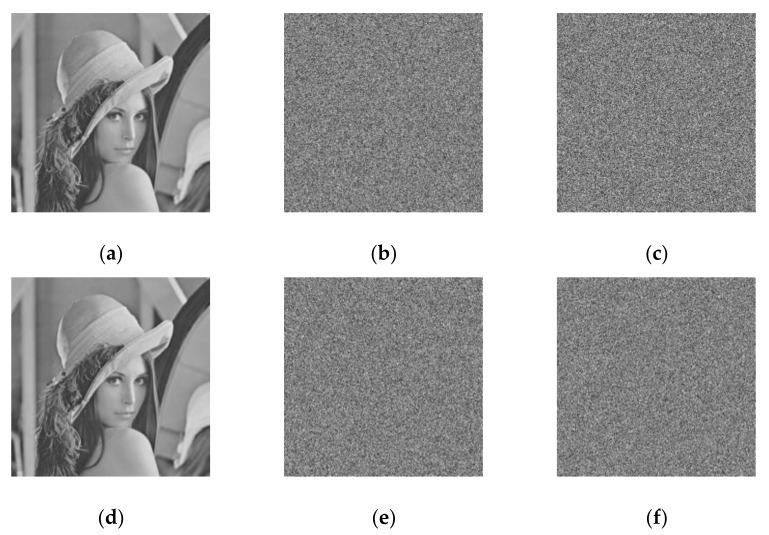
Key sensitivity analysis: (**a**) the plaintext image; (**b**) the ciphertext image $E_{1}$ encrypted by $K_{1}$; (**c**) the ciphertext image $E_{2}$ encrypted by $K_{2}$; (**d**) the decrypted image using correct key; (**e**) the decrypted $D_{1}$ from $E_{1}$ using $K_{2}$; (**f**) the decrypted $D_{2}$ from $E_{2}$ using $K_{1}$.

An image encryption scheme should firstly have a large enough key space to resist brute-force attacks. The scheme proposed in this paper has a key space of $2^{256}$ since the key length is 256 bits. Secondly, it is very sensitive to the initial key, otherwise the incorrect keys, which are slightly different from the initial key, can also obtain the plaintext information. Figure 11 shows the key sensitivity results. The same plaintext image is encrypted and decrypted by two keys $K_{1}$ and $K_{2}$ with one bit difference. Each key can decrypt the original image. If the other key is used for decryption, the original image information cannot be obtained. Thus, the proposed scheme is sensitive to its keys in both the encryption and decryption processes.

### 4.2. Histogram Analysis

Histograms can illustrate the distributions of pixel values of image. The histogram of the original and the encrypted images are presented in Figure 10. It can be clearly observed that the distributions of the encrypted images are random and very different from the distributions of the original images. When verifying the security of encrypted images, histogram analysis is necessary, but insufficient to verify the uniformity of encrypted images. In order to further evaluate the uniformity of the histogram of the encrypted images, we use the chi-square test in this paper. Its statistic $\chi^{2}$ value can be defined as:

(11) $$\chi^{2}=\sum_{i=0}^{255}\left(\frac{E_{i}-Z}{Z}\right),$$

where $E_{i}$ is value of the current pixel, and Z is the expected occurrence frequencies of each pixel. When α=0.05, $\chi_{0.05}^{2}=293.2478.$ A small $\chi^{2}$ value means the much more uniform distribution of the histogram of an image. The encryption image can pass the chi-square assessment when the calculated $\chi^{2}$ value of a ciphertext image does not exceed 293.2478 [28]. The chi-square values of virous encryption images are shown in Table 2. Obviously, all results do not exceed 293.2478, which shows that the distributions of the histogram of the encrypted images using the proposed encryption scheme are uniformly distributed.

### 4.3. Correlation Analysis

**Figure 12:**
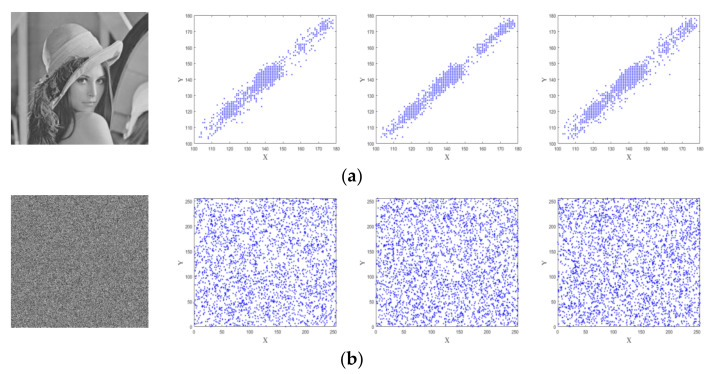
The correlation distributions: (**a**) the plaintext image and correlation distributions of three directions; (**b**) the ciphertext image and correlation distributions of three directions.

There is a strong correlation between each pixel of the digital image, which means that there is a small difference in the gray value between each pixel in a large area of the digital image. The pixel correlation of an image includes three directions: horizontal, vertical, and diagonal. One of the goals of an encrypted image is to reduce the correlation between adjacent pixels. The lower the correlation between pixels, the better the encryption algorithm, the better the encryption effect, and the higher the security. The correlation of two pixels sequences can be defined by:

(12) $$r_{uv}=\frac{\mathrm{cov}\left(u,v\right)}{\sqrt{D\left(u\right)}\;\sqrt{D\left(v\right)}},$$

(13) $$\mathrm{cov}\left(u,v\right)=\frac{1}{N}\sum_{i=1}^{N}\left(u_{i}-E\left(u\right)\right)\left(v_{i}-E\left(v\right)\right),$$

(14) $$D\left(u\right)=\frac{1}{N}\sum_{i=1}^{N}{\left(u_{i}-E\left(u\right)\right)}^{2},$$

(15) $$E\left(u\right)=\frac{1}{N}\sum_{i=1}^{N}u_{i},$$

where u and v are adjacent pixels values, and $r_{uv}$ is the correlation coefficient of the adjacent pixels. When $r_{uv}\to 1,$ which indicates that adjacent pixels are highly correlated, and when $r_{uv}\to 0,$ which demonstrates that adjacent pixels are low correlated [33]. In other words, when testing the relationship number of the phase encrypted image, the closer the value is to 0, the lower the correlation is. The 3000 pairs of adjacent pixels, from the original and encrypted images of three directions in horizontal, vertical, and diagonal directions are randomly selected. The distributions of these pairs are shown in Figure 12. As Figure 12 illustrates, the pixels of the plaintext image are close to the diagonal line, while the pixels of the ciphertext image are randomly distributed. Table 3 presents the comparison results of the correlations of adjacent pixel from plaintext and ciphertext images. Here, we use Lena with size of 512×512. It can be observed that the $r_{uv}$ values of the proposed method are closer to 0 compared to the other schemes.

### 4.4. Differential Attack

Diffusion is an important property in the process of image encryption. When the pixel position or value of the original image changes a little, this change will spread to the whole image in an unpredictable way under the diffusion operation. In general, the attacker will modify one or several pixels in the original image, and then observe the changes of the results to find some meaningful relationship between the original image and the encrypted image. A good encryption algorithm, if a small change in the original image causes a great change in the scrambling and diffusion effect of the encrypted image, then the efficiency of differential attack is relatively low. In order to evaluate the ability of an image encryption scheme to resist differential attack, we use the number of pixels change rate (NPCR) and unified averaged changed intensity (UACI) tests [35]. Suppose that $C_{1}$ and $C_{2}$ represent two encrypted images, respectively. NPCR and UACI can be described as follows:

(16) $$NPCR=\frac{\sum_{m=1}^{M}\sum_{n=1}^{N}D\left(m,n\right)}{MN}\times 100\%,$$

(17) $$D\left(m,n\right)=\left\{\begin{array}{c} 1\;\;for\;C_{1}\left(m,n\right)\neq C_{2}\left(m,n\right)\\ 0\;\;\;\;\;\;\;\;otherwise\;\; \end{array}\right.,$$

(18) $$UACI\left(C_{1},C_{2}\right)=\sum_{m=1}^{M}\sum_{n=1}^{N}\frac{\left|C_{1}\left(m,n\right)-C_{2}\left(m,n\right)\right|}{255\times M\times N},$$

where $C_{1}$ and $C_{2}$ are two encrypted images, whose original images have only one pixel change, and $D\left(m,n\right)$ represents the number of different pixels of the encrypted images $C_{1}$ and $C_{2}.$ The ideal expectations of NPCR and UACI are $NPCR_{E}=99.6094$ and $UACI_{E}=33.463507,$ respectively [35]. In this test, one pixel from each original image is randomly chosen, and its value is changed to generate another original image. The mean values of NPCR and UACI of serval encryption schemes are shown in Table 4.

Here, we use six color images with a size of 512×512 in USC-SIPI Miscellaneous dataset as examples. The NPCR and UACI test values of the ciphered images are presented in Table 4. The mean values of NPCR and UACI of serval encryption schemes are shown in Table 5. Obviously, compared with the results of other references, the synthesis results of the algorithm in this paper are closer to the ideal expected value, which shows that the proposed algorithm has better effect in resisting differential attack.

### 4.5. Two-Dimensional Detrending Fluctuation Analysis

In order to implement the scaling analysis of the different encryption schemes, the Two-Dimensional Detrending Fluctuation Analysis (2D-DFA) [37] is used to analyze the original image P, the confusion image F, and the diffusion image E when the scrambling stage considers two operations in this paper. Table 6 presents the result of the scaling analysis for encrypting the same image with different schemes, where α is the scaling fluctuation exponent. When the scaling fluctuation exponent α of the ciphertext image is close to 1, we assume that the encryption system is secure from a perception point of view and does not reveal any information of the original image [38]. As can be seen from Table 6, the values of the scaling exponents of diffusion image E are lower than that of the plaintext image P, and they are close to 1. Therefore, the encrypted image does not reveal any information that can distinguish the original image.

## 5. Conclusions

In this work, a 3D chaotic system with high complexity, called the 3D-ICM, was proposed. The excellent hyperchaotic dynamic behavior of the system has been described via quantitative evaluation criteria, such as LE and SE. Furthermore, compared with the existing chaotic system, it can be seen that the 3D-ICM has superior chaotic characteristics, which makes it usable in the field of image encryption. Thus, we proposed a chaotic image encryption scheme based on confusion and diffusion and used the 3D-ICM as a chaotic sequence generator. The scheme has low time complexity because it only involves one multiplication operation in the diffusion process. In addition, the scheme can also resist various attacks including brute-force attack, statistic attack, and differential attack, so it has a high level of security. In the future work, we will investigate the further application of the scheme in video encryption and field-programmable gate array (FPGA).

## Figures and Tables

**Table 1:** The time (second) required to encrypt images using different schemes.

Schemes	128×128	256×256	512×512	1024×1024
Diaconu [33]	0.0567	0.2014	0.9731	3.8377
HZ [34]	0.1335	0.5783	2.4913	9.9185
ZBC1 [35]	0.0796	0.3034	1.4824	5.8175
XLLH [36]	0.0212	0.1019	0.4924	20.144
Proposed method	0.0171	0.0304	0.1314	0.7021

**Table 2:** The $\chi^{2}$ distribution results of encryption image using the proposed method.

Images	Lena	Gray	Ruler	Boat	Pepper
$\chi^{2}$	252.0624	234.4568	227.3544	226.3549	241.9653

**Table 3:** Adjacent pixel correlations of the plaintext image “Lena” and its ciphertext image using different encryption schemes.

Schemes	Horizontal	Vertical	Diagonal
“Lena” image	0.9400	0.9769	0.9567
DS [9]	−0.0068	−0.0062	0.0070
HZ [34]	0.0034	−0.0010	−0.0002
XLLH [36]	0.0003	0.0014	0.0022
ZBC1 [35]	−0.0054	0.0042	0.0032
LSZ [37]	−0.0015	−0.0021	0.0019
Proposed method	−0.0009	−0.0012	0.0010

**Table 4:** The NPCR and UACI test values of ciphered images.

Images	NPCR (%)			UACI (%)		
	R	G	B	R	G	B
4.1.01.tiff	99.6189	99.6108	99.6098	33.4652	33.4636	33.4507
4.1.03.tiff	99.6139	99.6201	99.6149	33.4982	33.4678	33.4789
4.1.04.tiff	99.6246	99.6154	99.6098	33.4532	33.4726	33.4592
4.2.03.tiff	99.6052	99.6209	99.6134	33.4585	33.4677	33.4728
4.2.07.tiff	99.6357	99.6258	99.6072	33.5240	33.4584	33.4601
Lena	99.6145	99.6254	99.6275	33.4612	33.4612	33.4704

**Table 5:** Comparison of the NPCR and UACI mean values of these images.

Schemes	NPCR			UACI		
	R	G	B	R	G	B
HZ [34]	99.5972	99.6072	99.6120	33.4649	33.4650	33.4652
ZBC1 [35]	99.6109	99.6139	99.6079	33.4631	33.4636	33.4641
XLLH [36]	99.6246	99.6106	99.6123	33.4644	33.4651	33.4650
LSZ [25]	99.6052	99.6152	99.6105	33.4749	33.4647	33.4652
Proposed method	99.6188	99.6179	99.6138	33.4767	33.4652	33.4654

**Table 6:** Comparison of the scaling exponents of different encryption schemes.

α					
Images *P*	Image P	Image E			
		ZBC1 [35]	XLLH [36]	LSZ [25]	Proposed Method
lena	2.1463	1.1365	1.2194	1.2984	1.0015
boat.5.12	2.1954	1.2365	1.1984	1.1954	0.9989
gray.21.512	1.9978	0.9826	0.9907	0.9976	1.0149
ruler.512	2.0084	1.2654	1.0689	1.0554	1.0023
elaine.512	2.3684	1.2748	1.1607	1.1747	1.0114

## Data Availability

All results and data obtained can be found in open access publications.
